# A New Test Detects Selection for Early Flowering in a Much-Studied Weed

**DOI:** 10.1371/journal.pbio.0040171

**Published:** 2006-04-25

**Authors:** Liza Gross

Plants use a wide range of reproductive strategies to get around the fact that they can't pull up stakes when conditions deteriorate. The life cycle of flowering plants precisely tracks local daylight and temperature cycles to optimize flowering and boost reproductive success. The timing of flowering is crucial. As any temperate-gardener knows, a delicate plant that flowers too early in the season is doomed to perish with the next frost.

Much has been learned about the genes plants use to synchronize flowering with favorable environmental conditions by studying *Arabidopsis thaliana*. This slight mustard weed grows throughout the Northern Hemisphere. Reflecting adaptations to local environments, plants from different locations flower at different times when grown under the same light and temperature conditions. In particular, many plants take a very long time to flower unless induced to do so by prolonged exposure to cold in a process known as vernalization (the same process that forces spring bulbs into early bloom). This requirement for vernalization has been linked to the *FRIGIDA (FRI)* gene, based on observations that plants with nonfunctional *FRI* variants, or alleles, flower early without forcing. Two of these loss-of-function alleles— *friCol* and *friLer*—are linked to many of the early-flowering plants found in Europe.

There's a good chance that a gene associated with a trait directly related to reproductive success would show signs of selective pressure—and that's just what a new study shows. Christopher Toomajian, Magnus Nordborg, and their colleagues developed a novel genomics-based approach to detect selection and provide evidence that the two *FRI* alleles are under selection for rapid flowering in *Arabidopsis*.

A standard approach for detecting selection on a particular genomic region relies on the theoretical predictions of the neutral theory of molecular evolution. The problem with this approach, Toomajian et al. argue, is that many other forces besides selection can cause a deviation in the data from what is expected under simple neutral models. And with the plethora of genomic polymorphism data, they explain, it's possible to forgo the models and compare genome-wide patterns of variation instead. If the pattern at a region of interest differs radically from the genomic pattern, the region may be under selection.

A 2002 study of polymorphism around the *FRI* locus found that plants carrying one of the early flowering alleles also shared long blocks of identical chromosomal regions, or haplotypes. Building on those results, the researchers looked for patterns of haplotype sharing in genomic data from 96 *Arabidopsis* plants to see if the length of these haplotypes was typical, in which case the region probably wasn't under selection, or unusual, in which case it probably was.

To compare haplotype sharing around the *FRI* alleles with sharing at thousands of other loci, the researchers developed a new test, called the pairwise haplotype sharing (PHS) score. This score includes a function that controls for population structure: since pairs of individuals from the same population are more closely related than those from different populations, they're more likely to share long haplotypes and could bias the results.

PHS scores were calculated for all alleles found in the dataset, including the two *FRI* alleles, which had abnormally high haplotype sharing scores compared with the “vast majority” of alleles. Thirty-one other alleles also showed higher-than-average PHS scores, which the researchers plan to investigate as possible selection candidates in a future study. They also measured haplotype sharing using another test developed by Pardis Sabeti and colleagues, called extended haplotype homozygosity, which relies on the relationship between an allele's population frequency and its linkage to surrounding loci to determine its likelihood of being under selection given its age. This measure, they show, identifies multiple alleles from very similar sets of individuals that tend to come from the same population. This property can erroneously attribute haplotype sharing caused by population structure to selection. But this potential drawback shouldn't be a problem for organisms with a less complex population structure, like humans.

The researchers acknowledge that scanning the genome for haplotype sharing can't pinpoint the target of selection, but these results strongly suggest an adaptive role for the *FRI* alleles. While the evidence for selection is stronger for *friCol* than for *frILer*, it may be that selection on *frILer* happened much earlier, since both alleles have similar effects on the plant. This possibility is supported by the estimated age of the alleles, which the researchers place at 800 generations ago for *friCol* and 3,200 generations ago for *frILer*. Thus, if one assumes at least one generation per year, the loss-of-function *FRI* alleles emerged on the heels of the last glacial retreat, some 13,000 years ago. The researchers speculate that the evolution of these early flowering alleles, which turn an annual weed into a rapid reproducer, reflects selection for “weediness” in response to agriculture—an intriguing possibility that future studies can explore.

## 

**Figure:**
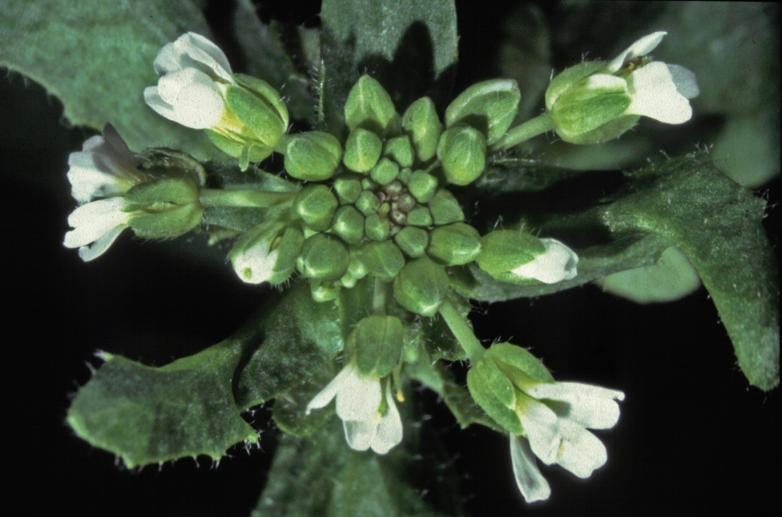
Gene variants that allow *Arabidopsis* plants to flower early in the season without “forcing” turn this annual weed into a rapidly reproducing superweed.

